# Hemolytic versus malproductive anemia in large granular lymphocytic leukemia

**DOI:** 10.1038/s41375-024-02323-6

**Published:** 2024-07-09

**Authors:** Olisaemeka Ogbue, Tariq Kewan, Carlos Bravo-Perez, Serhan Unlu, Naomi Kawashima, Nakisha D. Williams, Arooj Ahmed, Luca Guarnera, Carmelo Gurnari, Valeria Visconte, Jaroslaw P. Maciejewski

**Affiliations:** 1 Department of Translational Hematology and Oncology Research, Taussig Cancer Institute, Cleveland Clinic, Cleveland, OH USA; 2 Department of Hematology and Oncology, Yale university, New Haven, CT USA; 3 Department of Hematology and Medical Oncology, Hospital Universitario Morales Meseguer, University of Murcia, IMIB-Pascual Parrilla, CIBERER - Instituto de Salud Carlos III, Murcia, Spain; 4 Department of Biomedicine and Prevention, University of Rome Tor Vergata, Rome, Italy

**Keywords:** Haematological diseases, Cancer

## To the Editor:

Large granular lymphocytic leukemia (LGLL) can be defined as a chronic clonal proliferation of either cytotoxic T- (CTLs) or natural killer (NK) cells (T- or NK-LGLL) [1]. In a typical clinical situation, the disease affects elderly patients, who may present with neutropenia (39–62%) and/or anemia (up to 50%) [1], but also with a variety of autoimmune diseases [2]. Particularly, while commonly anemia in the context of LGLL is malproductive and reticulocytopenic resembling pure red cell aplasia (PRCA) [3, 4], we have also occasionally encountered cases with increased/preserved reticulocyte counts. A clinical workup done on these patients revealed either immune hemolytic anemia (IHA), hypersplenism or both. Thus, the pathophysiologic underpinnings of anemia in LGLL can be diverse. Because clinical distinction has therapeutic consequences and may provide clues to the pathogenesis of LGLL, we sought to investigate the features of hemolytic versus reticulocytopenic anemia by conducting a comprehensive and systematic study of hemolysis occurring in the context of a large cohort of patients affected by this disease.

We analyzed clinical and molecular features of 262 patients diagnosed (median follow up: 84 months) at The Cleveland Clinic Foundation (CCF) from 1998 to 2022 to identify those experiencing hemolysis. LGLL diagnosis was based on the presence of ≥4/6 criteria: i) chronically elevated LGL count (>0.5 × 10^9^/dL over >3 months), ii) clonal TCR rearrangement, iii) VB expansion, iv) flow cytometric detection of aberrant CTL or NK-cell proliferation, v) *STAT3/5* mutation and vi) LGL infiltration of the marrow [5]. IHA diagnosis was based on the evidence (at any time during clinical course) of signs of hemolysis namely reticulocytosis (>2% and/or >60 ×10^3^/uL), elevated LDH levels, and low haptoglobin. Conversely, anemia characterized by a reticulocyte index (RI) of <2 without evidence of hemolysis was classified as malproductive/hyporegenerative. Hemoglobin (Hb) response criteria including complete response (CR) and partial response (PR) were determined using consensus definitions [6]. Transfusion dependence was defined as requirement of ≥2 units of red blood cell (RBC) every 2 weeks for at least 3 months due to Hb <7 or symptomatic anemia. Fisher’s exact test was used to determine statistical significance at *p* < 0.05.

**Fig. 1:**
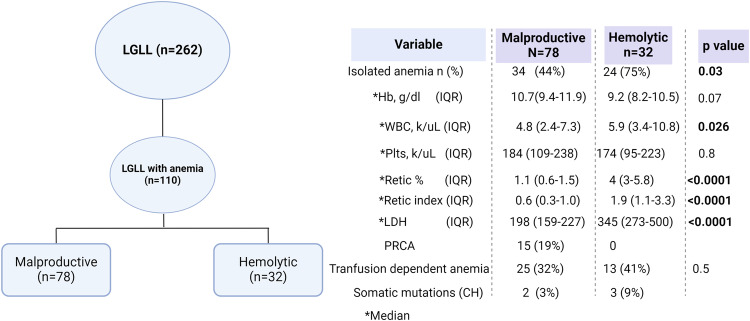
Categorization of anemia associated with large granular lymphocytic leukemia. Left panel: Indication of the number of patients in the study cohort. Right Panel: Number of patients with malproductive versus hemolytic anemia.

Overall, the median age at diagnosis was 63 years (interquartile range [IQR]: 55–72) with a M:F ratio of 0.5. Majority of patients had a diagnosis of T-LGLL (*n* = 236), whereas 26 (10%) had NK- LGLL (Supplementary Table S1). Of all cohort, *n* = 110 (42%) were identified to have anemia related to LGLL (Fig. 1). There were 78 anemic patients with RI < 2, among whom 19% (15/78) had PRCA. In 32/262 cases (12%), sufficient clinical and laboratory features were found to firmly establish the diagnosis of IHA (Supplementary Table S2). Of these, 22 (68%) had T-LGLL and 8 (25%) had NK-LGLL. IHA was more likely to present as an isolated anemia in 75% (*n* = 24) of cases (*p* = 0.003) while the remaining 8 (25%) presented either as bicytopenia (*n* = 5; 4/5 neutropenia) or pancytopenia (*n* = 3). Other parameters which distinguished hemolytic from malproductive anemia in LGLL included a lower median Hb nadir, g/dL (7.3 vs. 10.7, *p* < 0.0001), higher median reticulocyte count %/index (4/1.9 vs. 1.1/0.6) and higher LDH, U/L (345 vs.198) (Fig. 1).

**Fig. 2:**
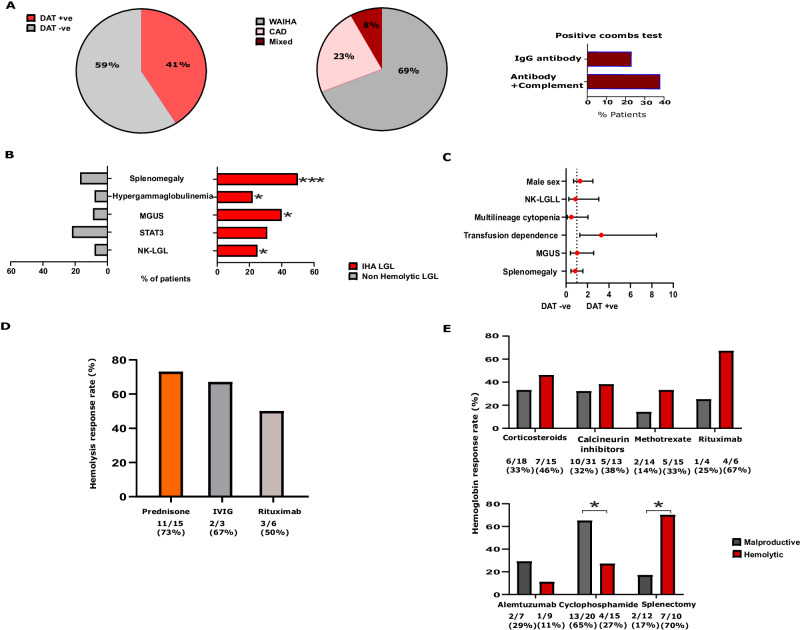
Clinical associations in our cohort. **A** Characteristics of Immune Hemolytic Anemia in Large Granular Lymphocytic Leukemia. **B** Contrasts Between Immune Hemolytic Anemia in LGLL (IHA LGLL) and Non-Hemolytic LGLL **C** Univariate Analysis Comparing Clinical Features of IHA LGL Based on Direct Anti-Globulin Test (DAT). **D** Hemolysis Treatment Response Rates in Immune Hemolytic Anemia Large granular lymphocytic leukemia. **E** Comparative Treatment Response Rates Between Malproductive and Hemolytic Anemia in Large Granular Lymphocytic Leukemia. Fisher’s exact test was used to determine significance at *p* < 0.05.

In IHA cohort, *STAT3* mutations were present in 10/32 cases (32% of T-LGLL and 38% of NK- LGLL). The direct anti-globulin test (DAT) was positive in 41% (*n* = 13/32) of IHA cases (Fig. 2A). Among DAT(+) patients, warm antibody IHA was observed in 9/13 (69%), cold agglutinin disease in 3/13 (23%) and one patient had mixed-antibody IHA. Among 8 patients with IgG AIHA, 5 were also positive for C3b and C3d antibodies. Hypergammaglobulinemia was observed in 7/32 IHA cases (22%), while an equal number presented with either primary (common variable immunodeficiency) or secondary (iatrogenic) immunodeficiency, characterized by low immunoglobulin levels. Other hematologic conditions included chronic B-cell dyscrasias such as monoclonal gammopathy (13/32) and chronic lymphocytic leukemia (4/32). Autoimmune connective tissue disorders such as rheumatoid arthritis co-existed in 4/32 patients. Clonal hematopoiesis was found in 9% of patients (Supplementary Table S3).

When comparing IHA LGLL with the remainder of the cohort, our analysis revealed a greater incidence of NK-LGLL cases among IHA patients (25% vs. 8%, *p* = 0.01), higher rates of *STAT3* mutations (31% vs. 22%, *p* = 0.08), MGUS (40% vs. 9%, *p* = 0.0001), and polyclonal hypergammaglobulinemia (22% vs. 8%, *p* = 0.02) (Fig. 2B). Of clinical importance is that splenomegaly was more likely to be present in IHA LGLL (50% vs. 17%, *p* = 0.0001). There was no difference in gender distribution, T- vs. NK-LGLL diagnosis, the presence of multilineage cytopenia, splenomegaly or MGUS between DAT(+) vs. DAT(−) patients (Fig. 2C). However, DAT(+) cases were more likely transfusion-dependent (69% vs. 21%, RR 3.3 [1.3–8.4], *p* = 0.01) and received 13 vs. 6 RBC transfusions per 10 patient/years. Bone marrow (BM) analysis revealed hyper/normocellular marrow in all IHA LGLL patients with 13% (4/32) showing erythroid hyperplasia.

In our retrospective analysis, strategies to alleviate anemia were targeted either to hemolysis and LGLL (*n* = 24) or LGLL alone (*n* = 8). Suppression of hemolysis as indicated by hemolytic indices was successful using steroids in 73% (11/15), whereas intravenous immunoglobulin (IVIG) or rituximab, were effective in 67% (2/3) and 60% (3/6) of cases, respectively (Fig. 2D). However, this corresponded to a Hb response in only 7/15 IHA cases treated with steroids, in 2/6 with rituximab and 1/3 with IVIG. When applying LGLL-directed therapy to patients with concomitant IHA, the overall Hb response rate (RR) was 27% (4/15) with cyclophosphamide achieving CR in 1 case PR in 3 cases, with a median duration of response of 47 months (IQR: 19–72). Subsequent lines of LGLL-directed therapies included calcineurin inhibitors like cyclosporine (CSA) or tacrolimus, showing a RR of 38% (5/13) with median duration of 72 months, methotrexate with a RR of 33% (5/15) with median duration of 21 months, and alemtuzumab with 11% RR (1/9) (Fig. 2E). Notably, LGLL-directed therapies improved hemolysis in only one patient with IHA treated with CSA.

Comparatively, oral cyclophosphamide resulted in a significantly stronger Hb response in malproductive than in IHA cases (65% vs. 27%, *p* = 0.04). In contrast, splenectomy when performed for LGLL refractory to medical therapy showed better outcomes in IHA than malproductive cases (70% vs. 17%, *p* = 0.03) (Fig. 2E). Among the 10 IHA patients who underwent splenectomy, 6 achieved CR, and one achieved PR (Supplementary Fig. S1A) accompanied by a significant improvement in Hb levels at one year post surgery (7.6 [IQR: 7–8] vs. 13.5 [13–14.3], *p* < 0.0001). Notably, post splenectomy responses remained durable in 5 out of 7 cases at a median follow-up of 87 months (IQR: 43–189) after the procedure (Supplementary Fig. S1B).

A significant portion of LGL patients presents with anemia and low reticulocyte counts indicating immune-mediated erythropoiesis inhibition by LGLL, akin to mechanisms in disease-associated neutropenia, which may coincide with other autoimmune phenomena [1, 7, 8] Although LGLL associated anemia is often accompanied by other cytopenias, previous reports have described isolated anemia in context of LGLL [3, 9]. The classification of LGLL-associated anemia according to bone marrow compensation capacity (indicated by reticulocyte counts), whether hemolytic or reticulocytopenic, holds clinical significance. Our study has uncovered a significant correlation between IHA and LGLL, indicating that this association is more common than previously thought [10]. However, there may be instances of overlap between these forms of LGLL-associated anemia, making it challenging to determine the predominant mechanism. Indeed, pure IHA with sufficient bone marrow response was observed in 13/32 (41%) IHA/LGLL patients, while others exhibited inadequate reticulocyte compensation, indicating possible coexisting bone marrow/erythroid suppression. Overall IHA LGLL often presented as an isolated anemia.

This study underscores the common occurrence of monoclonal gammopathy in patients with LGLL [2, 11], along with splenomegaly and polyclonal hypergammaglobulinemia, particularly in cases involving IHA. The correlation between hypergammaglobulinemia and the severity of anemia suggests a humoral mechanism contributing to IHA and may also be responsible for the destruction of erythroid precursors in the maladaptive component of the disease. At this point it can only be stipulated whether a stronger association of IHA with NK-LGLL indicates the pathophysiologic role of Fc receptor, which is more abundantly expressed in NK-cells than in CTLs and its involvement in antibody-dependent cell-mediated cytotoxicity reactions in vivo [12, 13]. No enrichment in *STAT3* mutations was observed in IHA LGLL. Two patients with LGL IHA received Tofacitinib, an indirect STAT pathway inhibitor, but showed no improvement in either hemolysis or anemia, consistent with other studies [14].

It is conceivable that IHA shares a pathophysiological link with LGLL, but the processes progress independently. Resolution of hemolysis was achieved with classical IHA treatments such as prednisone, IVIG and rituximab, resulting in a reduction in transfusion burden for responders. However, our observations also suggest that cytopenias (e.g., anemia) with LGLL-associated hemolysis may require T/(NK)-cell directed drugs to establish a more complete or more lasting response.

Study limitations stem from the retrospective nature of our investigation and the limited number of patients within treatment subgroups. Despite these constraints, our findings underscore the therapeutic implications of differentially diagnosing LGLL-associated anemia (Supplementary Fig. S2). T-cell directed IST may be more suitable for malproductive anemia of LGLL, while classic anti-hemolytic therapies, including splenectomy for refractory cases [15], could be considered for LGLL with hemolysis. However, it is important to note that anemia even in the latter cases is likely multifactorial and consequently successful therapy of LGLL would be needed to prevent relapse and recurrence of cytopenias.

### Supplementary information

Supplementary File

## Data Availability

All data for reproducibility are present in the main text and supplemental material. Requests for additional information not provided in the main text or supplementary material should be sent to the corresponding author maciejj@ccf.org.
