# Supplementary material for: Hemolytic versus malproductive anemia in large granular lymphocytic leukemia
Source: Leukemia. 2024 Jul 9;38(8):1839–42. doi: 10.1038/s41375-024-02323-6 (PMC11286512; doi:10.1038/s41375-024-02323-6)
Supplement: Supplementary file 1 — Supplementary File [file 41375_2024_2323_MOESM1_ESM.docx]

# Baseline characteristics of our cohort

| **Variables** | **All (n=262)** | **Non-HA LGL (n=230)** | **LGL HA (n=32)** | **P value** |
| --- | --- | --- | --- | --- |
| **Female gender, n (%)** | 176 (67%) | 164 (71%) | 12 (38%) | **0.0001** |
| **Caucasian race, n (%)** | 130 (50%) | 106 (46%) | 24 (75%) | **0.001** |
| **Age, Years (IQR)*** | 63 (55-72) | 66 (56-73) | 60 (53-66) | 0.1 |
| **WBC, k/uL (IQR)*** | 5.1 (3-8.8) | 5.10 (3-8.6) | 6.3 (3.4-10.8) | 0.08 |
| **Hb, g/dL (IQR)*** | 11.7 (9.8-13.3) | 11.9 (9.95-13.35) | 9.2 (8.2-10.5) | **0.003** |
| **Plts, k/uL (IQR)*** | 190 (130-253) | 189 (131-244) | 174 (95-223) | 0.5 |
| **ANC, k/uL (IQR)*** | 1.5 (0.7-2.5) | 1.6 (0.7-2.7) | 1.2 (0.8-1.8) | 0.5 |
| **Abs Lymphocyte count (IQR)*** | 2.6 (1.3-4.4) | 2.6 (1.4-4.3) | 3.3 (1.8-5.8) | **0.0009** |
| **Multilineage/Pancytopenia (%)** | 60 (30%) | 52 (30%) | 8 (25%) | 0.8 |
| **Oligoclonal/Indeterminate TCR rearrangement** | 12 (5%) | 10 (5%) | 2 (6%) | 0.14 |
| **LGL Count (IQR)*x1000** | 1.4 (0.7-2.8) | 1.3 (0.7-2.6) | 2.4 (1.2-4.3) | **0.0003** |

*Median

WBC: White blood cell count; Hb: Hemoglobin; Plts: Platelets; ANC: Absolute neutrophil count; LGL: Large granular lymphocytosis

# Definitions in Immune hemolytic anemia^1^

| Immune hemolytic  anemia (IHA) | Anemia related to a reduction of red blood cell (RBC) lifespan due to increased destruction by antibodies directed against antigens on RBCs membrane. |
| --- | --- |
| Diagnostic  criteria for IHA | In this scenario, characterized by LDH levels exceeding the upper limit of normal (ULN) and haptoglobin falling below the lower limit of normal (LLN), hemolysis is confirmed with a positive direct antiglobulin test (DAT) and exclusion of alternative causes, including delayed hemolytic transfusion reaction. |
| DAT-negative IHA | Typically caused by non-IgG autoantibodies or RBC-bound antibody levels below the sensitivity threshold. The diagnosis requires evident hemolysis, exclusion of other  hereditary and acquired hemolysis causes. |
| Warm AIHA (wAIHA) | wAIHA is diagnosed in patients with a positive DAT, typically for IgG or C3d ±IgG when a clinically relevant cold reactive antibody has been excluded. |
| Cold agglutinin disease (CAD) | Diagnosed when DAT is strongly positive for C3d and weakly positive or negative for IgG with a Cold agglutinin (CA) titer of 64 or greater at 4°C. |
| Mixed AIHA | Mixed IHA is diagnosed in patients with a DAT positive for C3d and IgG, a cold antibody with a thermal amplitude ≥30^o^C and evidence of a warm IgG antibody by IAT or IAT eluate. |

**Somatic mutations associated with immune hemolytic anemia and large granular lymphocytic leukemia.**

| **Mutation** | **Position** | **VAF (%)** |
| --- | --- | --- |
| **ASXL1** | **p.R419*, c.1255A>T** | **41.6** |
| **IDH2** | **p.R140Q, , c.419G>A** | **43.1** |
| **IDH2** | **p.R172W, c.514A>T** | **38** |
| **KRAS** | **p.G12A, c.35G>C** | **1.8** |
| **NRAS** | **p.Q61K, c.181C>A** | **45** |
| **SRSF2** | **p.P95H, , c.284C>A** | **44.8** |

**A B**


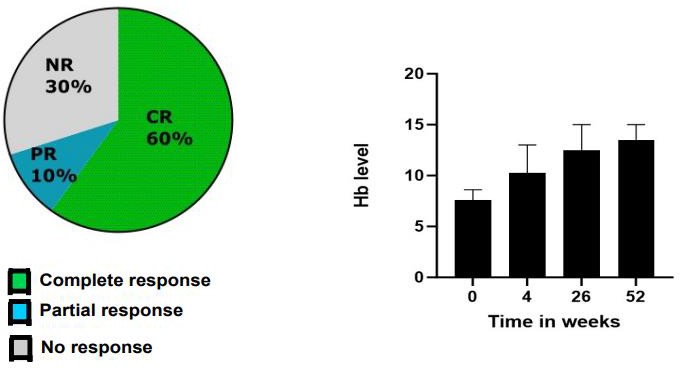


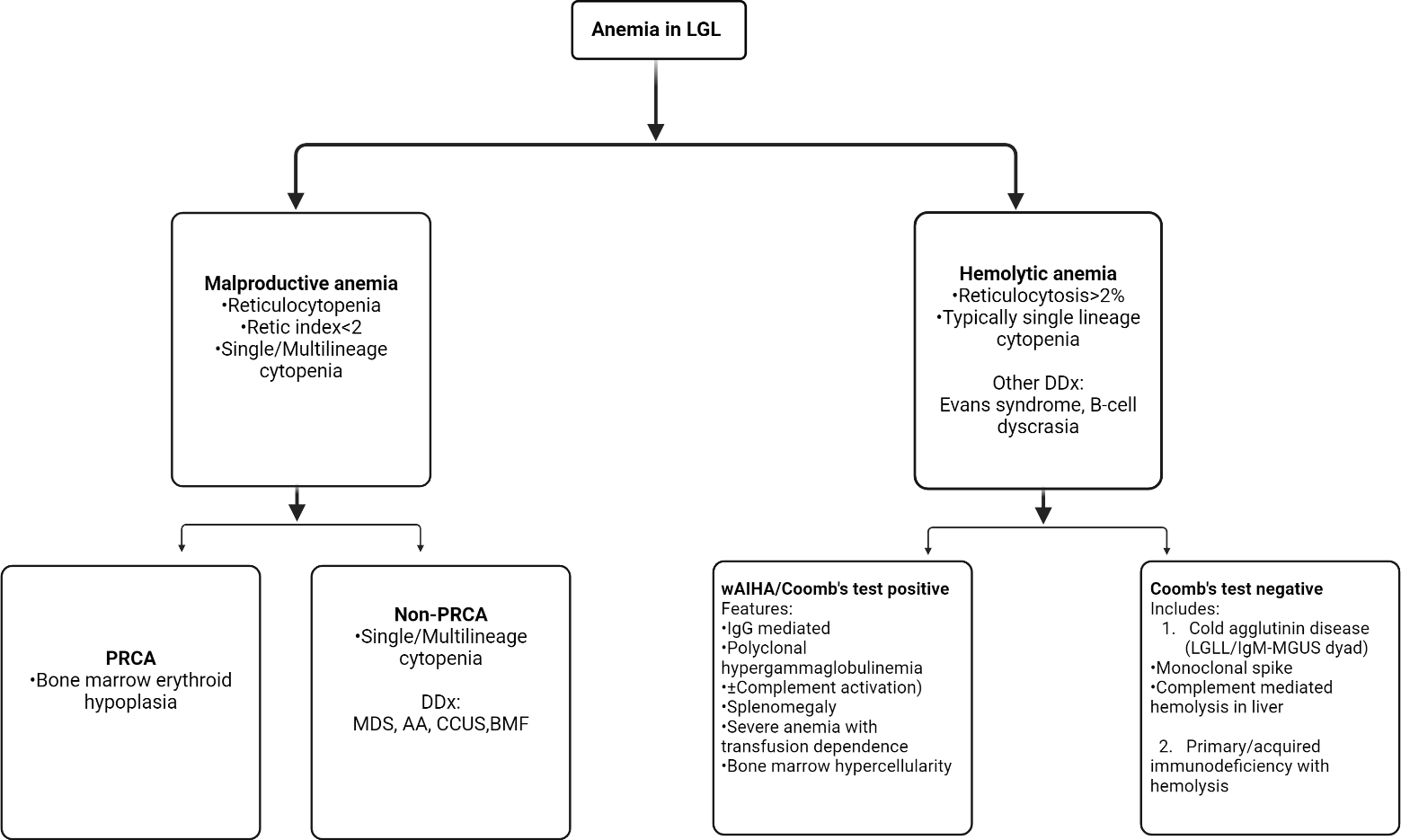


Supplementary figure legends:

**Supplementary figure 1**: Response to splenectomy in IHA LGLL

**A:** Type of response

**B:** Rise in hemoglobin (Hb) in weeks.

**Supplementary figure 2**: Differential diagnosis of Large granular lymphocytic leukemia associated anemia

References:

1. Jäger U, Barcellini W, Broome CM, Gertz MA, Hill A, Hill QA, et al. Diagnosis and treatment of autoimmune hemolytic anemia in adults: Recommendations from the First International Consensus Meeting. Blood Rev. 2020;41:100648. doi:10.1016/j.blre.2019.100648
